# Isolation and Validation of an Endogenous Fluorescent Nucleoid Reporter in *Salmonella* Typhimurium

**DOI:** 10.1371/journal.pone.0093785

**Published:** 2014-04-02

**Authors:** Ioannis Passaris, Anirban Ghosh, William Cenens, Chris W. Michiels, Jeroen Lammertyn, Abram Aertsen

**Affiliations:** 1 Laboratory of Food Micobiology, Department of Microbial and Molecular Systems (M2S), Faculty of Bioscience Engineering, KU Leuven, University of Leuven, Belgium; 2 BIOSYST-MeBios, Faculty of Bioscience Engineering, KU Leuven, University of Leuven, Belgium; Indian Institute of Science, India

## Abstract

In this study we adapted a Mu*d*-based delivery system to construct a random *yfp* reporter gene (encoding the yellow fluorescent protein) insertion library in the genome of *Salmonella* Typhimurium LT2, and used fluorescence activated cell sorting and fluorescence microscopy to screen for translational fusions that were able to clearly and specifically label the bacterial nucleoid. Two such fusions were obtained, corresponding to a translational *yfp* insertion in *iscR* and *iolR*, respectively. Both fusions were further validated, and the IscR::YFP fluorescent nucleoid reporter together with time-lapse fluorescence microscopy was subsequently used to monitor nucleoid dynamics in response to the filamentation imposed by growth of LT2 at high hydrostatic pressure (40–45 MPa). As such, we were able to reveal that upon decompression the apparently entangled LT2 chromosomes in filamentous cells rapidly and efficiently segregate, after which septation of the filament occurs. In the course of the latter process, however, cells with a “trilobed” nucleoid were regularly observed, indicative for an imbalance between septum formation and chromosome segregation.

## Introduction

The intracellular organization of prokaryotes is proving to be increasingly complex, often constituting a structural and functional prelude to eukaryotic counterparts and emphasizing that bacteria are useful models for studying universal cellular mechanisms. The nucleoid is one of the most important structures inside the bacterial cell, and although it has been first described 50 years ago, only very recently more insight was gained about its organization and mechanism of compaction [1], [2]. The chromosome is mainly compacted by negative DNA supercoiling which gives rise to supercoiled domains that are topologically insulated from each other [3]. The DNA rotation of these topological domains is restricted by so-called domainins, which have been proposed to include small nucleoid associated proteins (NAPs) [4], structural maintenance of chromosome (SMC) condensing complexes [5], topoisomerases [6], RNA polymerase and even RNA [7], [8]. Additional organization of the *E. coli* nucleoid is accomplished by large DNA regions (around 1 Mb in size), termed macrodomains, which restrict certain rearrangements in the linear-order sequence of the chromosome [9].

Although it is clear that more and more valuable information is obtained about the standing architecture of the nucleoid, studies involving global chromosome dynamics in response to environmental stress are generally lacking. Moreover, the use of specific DNA binding dyes (DAPI, bis-benzimides, …) and fixing procedures to fluorescently stain and image the bacterial nucleoid are often genotoxic and prone to produce artefacts. Such approaches are therefore not suitable to properly follow-up live nucleoid dynamics in cells, especially if the latter are already suffering DNA damage and/or affected in their DNA repairing capabilities.

In this study, we screened a random translational YFP library in *Salmonella* Typhimurium LT2 for chromosome based nucleoid reporters, using a customized transposon. Two nucleoid reporters were found of which one was further validated and subsequently used to track the nucleoid dynamics of LT2 after stressful growth under high hydrostatic pressure (HP), a cryptic but environmentally relevant stress that causes excessive filamentation in mesophilic bacteria [10].

## Materials and Methods

### Strains and growth conditions

Bacterial strains, phages and plasmids used throughout this study are listed in Table 1. For culturing bacteria, Lysogeny Broth (LB; [11]) medium was used either as broth or as agar plates after the addition of 15% (for spreading plates) or 7% (for soft-agar plates) agar. Cultures were grown in LB broth for 16–20 h at 37°C under well-aerated conditions (200 rpm on an orbital shaker) to reach stationary phase. Exponential phase cultures were in turn prepared by diluting stationary phase cultures 1/100 or 1/1000 in pre-warmed broth, and allowing further incubation at 37°C until an optical density at 600 nm (OD_630_) of 0.4–0.6 was reached. When appropriate the following chemicals (Applichem, Darmstadt, Germany) were added to the growth medium at the indicated final concentrations: ampicillin (100 μg/ml; Ap^100^), chloramphenicol (30 μg/ml; Cm^30^), kanamycin (50 μg/ml; Km^50^), tetracycline (20 μg/ml; Tc^20^), oxytetracycline (10 μg/ml; OxyTc^10^), glucose (0.2%) and L-arabinose (0.2%).

**Table 1:** Strains, phages and plasmids used in this study.

Name	Relevant characteristic	Source our reference
***Strains***		
*Escherichia coli*		
K-12 DH5α	F^−^ φ80*lacZ*ΔM15 Δ(*lacZYA-argF*) U169*endA1recA1hsdR17deoRthi1supE4412 gyrA96relA1*	Laboratory collection
K-12 MG1655	F^−^ λ^−^ *ilvG* ^−^ *rfb-50 rph-1*	[36]
*Salmonella* Typhimurium		
LT2	Parental strain	[37]
TH2145	LT2 *hsiD*1284::Mu*d*K *hisA*9944::Mu*d*I	Kelly Hughes (University of Utah, USA)
TH2145 Mu*d*Y	TH2145 in which *lacZYA-npt* has been replaced by *yfp-frt-cat-frt*	This work
LT2 *iscR*::Mu*d*Y	MudY transposon transposon integrated 433 bp from ATG (+1) of *iscR* yielding the IscR144::YFP protein	This work
LT2 *iscR*::*yfp*	C-terminal fusion of *yfp* to *iscR* with SGGGG linker, constructed via pAC	This work
LT2 *iscR*::*yfp recA1 srl-202*::Tn*10*	RecA deficient variant of LT2 *iscR*::*yfp*	This work
LT2 *iscR::yfp-frt-cat-frt*	C-terminal fusion of *yfp* to *iscR* with SGGGG linker, constructed via pGKBD	This work
TT521	LT2 *recA1 srl-202*::Tn*10*	John Roth (University of California at Davis, USA)
LT2 *iolR*::Mu*d*Y	MudY transposon transposon integrated 478 bp from ATG (+1) of *iolR* yielding the IolR159::YFP protein	This work
LT2 *iolR*::*yfp*	C-terminal fusion of *yfp* to *iolR* with SGGGG linker	This work
LT2K2	SOS-reporter strain of LT2	[31]
***Phages***		
P22 *HT105/1 int-201*	Integration deficient mutant of P22 used for generalized transduction	Kelly Hughes (University of Utah, USA)
λNK1323	λ phage used for mini-Tn*10* transposon mutagenesis of pKD46	[17]
***Plasmids***		
pKD46	Encodes Lambda red genes under control of arabinose inducible promoter	[16]
pKD46 *bla*::Tn*10*	Encodes Lambda red genes under control of arabinose inducible promoter and is OxyTc^10^ resistant instead of Amp^100^	This work
pCP20	Encodes Flp for recombining *frt* sites	[23]
pAc	*yfp-frt-cat-frt* template for recombineering of *yfp*. The *cat* gene is transcribed in the opposite direction compared to the *yfp* gene.	[18]
pGKBD	*yfp-frt-cat-frt* template for recombineering of *yfp.* The *cat* gene is transcribed in the same direction compared to the *yfp* gene.	Laboratory collection

For obtaining growth curves, stationary phase cultures were diluted 1/1000 in 300 μl of LB medium, placed in a honeycomb well and incubated in the Bioscreen C system (Thermo Labsystems OY, Helsinki, Finland) for a 24 h period at 37°C, with regular shaking and automatic OD_630 nm_ measurements every 15 min. The resulting OD_630 nm_ values were averaged across three replicate cultures and standard deviations were<11%.

Phages were propagated on *S.* Typhimurium LT2 as plaques in LB soft-agar or as lysates in LB broth as described previously [12]. Phage stocks were filter sterilized with 0.2 μm filters (Fisher Scientific, Aalst, Belgium) and chloroform was added to maintain sterility. Generalized transduction was performed with phage P22 *HT105/1 int-201* as described previously [12], [13]. This mutant is unable to integrate into the host chromosome as a prophage due to the lack of integrase (Int) activity. To discriminate phage infected from uninfected colonies, mint green plates (MG; [14] and http://rothlab.ucdavis.edu/Recipes/mintgreen.html) were used to indicate cell lysis. The latter medium contains glucose as a carbon source, and a pH indicator dye that turns dark green at sites where phage infection causes cell lysis and the concomitant release of organic acids.

### Construction of Mu*d*Y

Construction of Mu*d*Y was done by modifying the well characterized Mu*d*K transposon [15] using the λ red system [16]. The original *S. Typhimurium* strain (TH2145, Table 1) adapted in this study contains an ampicillin resistance gene in the Mu*d*I part, which makes it incompatible with pKD46 antibiotic selection. This prompted us to replace the *bla* gene in pKD46 with the tetracycline resistance cassette from the mini-Tn*10* transposon. For this, phage λNK1323 [17] was mixed with a stationary culture of *E. coli* K-12 MG1655 pKD46, and plated out on OxyTc^10^ plates. After overnight incubation, the plates were pooled and subsequently subjected to plasmid DNA extraction (GeneJET Plasmid Miniprep Kit, ThermoScientific). Next, this pool of plasmid DNA was transformed by electroporation to *E. coli* DH5α and transformants were first selected on OxyTc^10^ and then individually screened for loss of ampicillin resistance. As such, a pKD46 derivative could be isolated in which the *bla* gene was knocked-out by the mini-Tn*10* transposon (designated pKD46 *bla*::Tn*10*).

Subsequently, the Mu*d*K donor strain TH2145 was equipped with pKD46 *bla*::Tn*10* and recombineering [16] was performed to exchange the original *lacZYA-npt* part of Mu*d*K with a *yfp-frt-cat-frt* cassette. For this, the *yfp-frt-cat-frt* cassette was PCR amplified (Phusion DNA polymerase, ThermoScientific) from plasmid pAC [18] with primers yfp_cat_Fw and yfp_cat_Rev (Table 2). The resulting strain, now harboring the Mu*d*Y transposon, was purified and then grown in LB broth at 30°C to suppress transposase expression [15] and without OxyTc^10^ to cure the pKD46 *bla*::Tn*10* plasmid. The resulting strain was named TH2145 Mu*d*Y (now sensitive to OxyTc^10^ and Km^50^, and resistant to Cm^30^ and Amp^100^) and was used as the donor strain for constructing a random Mu*d*Y insertion library.

**Table 2:** Primers used in this study.

Primer name	Sequence (5′-3′)^a^
yfp_cat_Fw	TTCAAATGAAACAGATGTATTAATTACTGCTTTTTATTCATTACATGGGGATCCC**GCTAGCAAAGGAGAAGAACTTTTC**
yfp_cat_Rev	CTGATGGCGCAGGGGATCAAGATCTGATCAAGAGACAGGATGAGGATCGTTTCGCA**GATATCCTCCTTAGTTCCTA**
mudY_out_up	CATCTGTTTCATTTGAAGCGCG
Y_linker_primer	CTGCTCGAATTCAAGCTTCT
linker1	TTTCTGCTCGAATTCAAGCTTCTAACGATGTACGGGGACACATG
phosphorylated linker2	TGTCCCCGTACATCGTTAGAACTACTCGTACCATCCACAT
iscR_yfp_C-term_Fw	CCGCGCGCCAGCGGTCGTGCGCAGGACGCTATCGACGTTAAATTACGCGCT*AGCGGTGGCGGTGGC* **GCTAGCAAAGGAGAAGAACT**
iscR_yfp_C-term_Rev_pAC	CGCGGCGTTCACCGCATGAGGCCGCCAGAAGAGATGGCGTAATATTTTAA**TATCCTCCTTAGTTCCTA**
iscR_yfp_C-term_Rev_pGKBD	CGCGGCGTTCACCGCATGAGGCCGCCAGAAGAGATGGCGTAATATTTTAA**GTGTAGGCTGGAGCTGCTTC**
iolR_yfp_C-term_Fw	CCATGTGTCTGGCGCAAACGTTAGCGGTTTCACTGGCGCTGGCGACGGAG*AGCGGTGGCGGTGGC* **GCTAGCAAAGGAGAAGAACT**
iolR_yfp_C-term_Rev	GTTTCACCACAATGCCGATGATCGCTAAATACGATCATCGGCTTGTTTTT**TATCCTCCTTAGTTCCTA**

a When relevant primer attachment sites are indicated in bold. The linker region coding for SGGGG is shown in italic.

### Validation of Mu*d*Y

To ensure that the Mu*d*Y donor strain was still capable of producing random transposon insertion mutants, a high-copy plasmid of around 6 kb containing a P_BAD_ promotor and *bla* resistance cassette was mutagenized with Mu*d*Y. First, a phage P22 *HT105/1 int-201* lysate was made from the Mu*d*Y donor strain and subsequently mixed with a stationary phase culture of *S.* Typhimurium LT2 containing the high copy plasmid and incubated for 30 min at 37°C to permit phage adsorption. The mixture was subsequently plated out on LB Cm^30^ Ap^100^ agar plates and incubated overnight. The resulting library of around 10,000 mutants was scraped off the plates, pooled and subjected to plasmid DNA extraction (GeneJET Plasmid Miniprep Kit, ThermoScientific). Next, this pool of plasmid DNA was transformed by electroporation to *S.* Typhimurium LT2 and transformants were selected on Cm^30^ and Amp^100^. 10 transformants were picked up and the exact location of Mu*d*Y in the plasmid was determined using the protocol described below. YFP fluorescence could be induced in one transformant by adding 0.2% arabinose, indicating the *yfp* gene became inserted in frame and downstream of the P*_BAD_* promoter.

### Construction of random Mu*d*Y insertion library

The P22 *HT105/1 int-201* lysate of TH2145 *hsiD*1284::Mu*d*Y *hisA*9944::Mu*d*I was mixed with a stationary culture of *S.* Typhimurium LT2 acceptor strain and incubated for 30 min at 37°C to permit phage adsorption. Next, the mixture was diluted in LB broth complemented with 10 mM EGTA and plated out on LB Cm^30^ agar plates containing 1 mM EGTA and incubated at 37°C. EGTA was added to block adsorption of P22 and thereby prevent subsequent cycles of infection on the plate [19]. Transposon mutagenesis is accomplished using the method described by Hughes and Roth (1988) [15]. In short, when the Mu*d*Y transposon, which lacks natural transposition activity, is packed into phage P22 together with the transposase of the adjacent Mu*d*I element, it is able to randomly transpose into the bacterial genome. Due to the packaging constraints of P22 (±44 kb) Mu*d*Y and Mu*d*I can never transpose simultaneously, as a consequence the transposase is lost by degradation and segregation yielding single insertion mutants with Cm^30^ resistance.

The resulting Mu*d*Y insertion library constituted of approximately 25.000 mutants and was scraped off the plates using LB broth containing 10 mM EGTA. This large pool was subjected to one round of fluorescence activated cell sorting (FACS).

### Fluorescence activated cell sorting

The Mu*d*Y pool in LT2 was sorted by a Fluorescence Activated Cell Sorter (FACS; BD influx cell sorter) to enrich for YFP expressing mutants. A 488 nm excitation laser in combination with a 530/40 nm emission filter was used, and the 0.1% most YFP-fluorescent clones were sorted and plated out on MG agar plates containing Cm^30^ to discern P22 infected from phage free Mu*d*Y mutants.

### Time-lapse fluorescence microscopy

Fluorescence microscopy was used to screen 200 mutants for nucleoid reporter activity. All fluorescence microscopy and time-lapse fluorescence microscopy experiments were performed with a temperature controlled (Okolab Ottaviano, Italy) Ti-Eclipse inverted microscope (Nikon, Champigny-sur-Marne, France) equipped with a TI-CT-E motorized condenser, a YFP filter (Ex 500/24 nm, DM 520 nm, Em 542/27 nm), a DAPI filter (Ex 377/50 nm, DM 409 nm, Em 447/60), and a CoolSnap HQ2 FireWire CCD-camera. For imaging, cells were grown to mid-log phase and placed between LB agar pads and a cover glass, essentially as described previously [20], and incubated at 37°C. Where appropriate, DAPI was added in the LB agar pad at a final concentration of 1 μg/mL, chloramphenicol at a final concentration of 2 μg/mL, nalidixic acid at a final concentration of 150 μg/mL and rifampicin at a final concentration of 100 μg/mL. Mitomycin C was added to the liquid culture 30 min prior to imaging at a final concentration of 1 μg/mL.

Images were acquired using NIS-Elements (Nikon) and resulting pictures were further handled with open source software ImageJ (downloaded from http://rsbweb.nih.gov/ij/).

### Mapping of Mu*d*Y insertions

Mapping of the Mu*d*Y insertions was performed in analogy with the method used by Kwon and Ricke (2000) [21]. First, 20 μL of linker1 (350 ng/μL) (see Table 2) was added to 18 μL of phosphorylated linker2 (350 ng/μL) and heated for 2 min at 95°C, after which the mixture was left to cool down and allow annealing of the linkers (Y linker). Genomic DNA of a Mu*d*Y mutant was extracted via phenol∶chloroform extraction [22] and completely digested with NlaIII (ThermoScientific). The digested DNA was purified with the GeneJET PCR Purification kit (ThermoScientific) and approximately 40 μg was ligated to 1 μg of the Y linker with 1 μL of T4 DNA ligase (1 unit/μL; ThermoScientific) in a final volume of 20 μL. After overnight incubation at 22°C the reaction mixture was heated at 65°C for 10 min to denature the ligase. 2 μL of this mixture was used as DNA template in a PCR mixture together with a primer specific to Mu*d*Y (Mu*d*Y_out_up), a primer specific to the Y linker (Y linker primer) and a Taq polymerase (DreamTaq DNA polymerase, ThermoScientific). The PCR products were purified, sequenced and the exact position of the Mu*d*Y transposon was determined using BLAST search. Initially, five clones were retained as nucleoid reporter candidates but after mapping the Mu*d*Y transposon it was found that this number could be reduced to two different clones (*iscR*::Mu*d*Y and *iolR*::Mu*d*Y). This clonal enrichment was likely due to the pooling of the library before sorting the most fluorescent cells.

### Construction of *iscR*::*yfp* and *iolR*::*yfp*

Based on the Mu*d*Y insertions in *iscR* and *iolR*, *yfp* translational fusions were *de novo* constructed at the 3′ end of the same genes. For this, the *yfp-frt-cat-frt* cassette was PCR amplified (Phusion DNA polymerase; ThermoScientific) from plasmid pAC [18] with primers iscR_yfp_C-term_Fw (Table 2) and iscR_yfp_C-term_Rev_pAC, and iolR_yfp_C-term_Fw and iolR_yfp_C-term_Rev, respectively. The obtained amplicons were subsequently used to generate LT2 *iscR*::*yfp* and LT2 *iolR*::*yfp* via recombineering in LT2 and flipping out the *cat* cassette, using pKD46 [16] and pCP20 [23], respectively. In the latter strains, the YFP moiety is C-terminally fused to the IscR or IolR protein, respectively, with a SGGGG linker. The LT2 *iscR*::*yfp recA1* strain was subsequently constructed by cotransducing the *recA1* and *srl-202*::Tn*10* alleles from TT521 (kindly provided by John Roth, University of California at Davis, USA) with P22 *HT105/1 int-201* [13] to LT2 *iscR*::*yfp*.

To mitigate the apparent growth defect of the LT2 *iscR*::*yfp* strain constructed via pAC, an LT2 *iscR*::*yfp-frt-cat-frt* strain was constructed by recombineering an *yfp-frt-cat-frt* amplicon obtained from plasmid pGKBD (Govers and Aertsen, unpublished) by PCR with primers iscR_yfp_C-term_Fw and iscR_yfp_C-term_Rev_pGKBD. In contrast to pAC, pGKBD contains an *yfp-frt-cat-frt* cassette in which the *cat* locus is oriented in the same direction as the *yfp* gene. In the latter strain, the YFP moiety is C-terminally fused to the IscR protein with a SGGGG linker.

### High pressure treatment

For treatment with HP, stationary phase cultures were 1/100 diluted in LB broth, after which 200 μl of this suspension was heat sealed in a sterile polyethylene bag after exclusion of the air bubbles, and subjected for 16-20 h to a pressure of 40–45 MPa in an 8 ml pressure vessel (HPIU-10000, 95/1994; Resato, Roden, The Netherlands), held at 37°C. After pressure release, cells were 1/10 diluted in fresh LB and quickly prepared for imaging as described above.

## Results

### Construction and validation of Mu*d*Y

**Figure 1:**
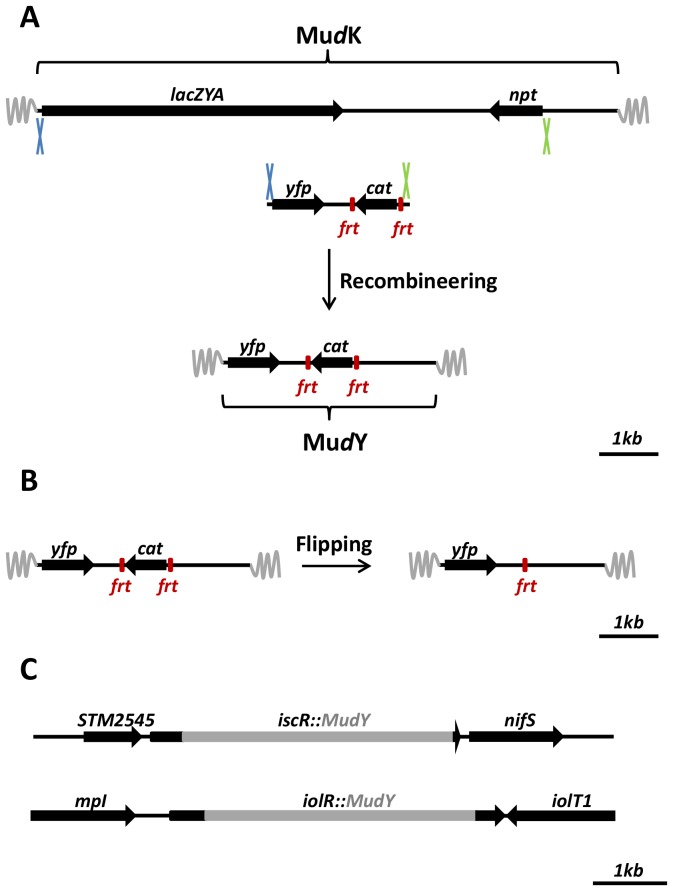
Construction and use of the MudY transposon. (A) During construction of the Mu*d*Y transposon, the *lacZYA*-*npt* of Mu*d*K was replaced by an *yfp*-*frt*-*cat*-*frt* module through recombineering (blue and green crosses indicate the homologous regions involved in recombination). (B) The *frt*-*cat*-*frt* cassette can be readily flipped out using the Flp recombinase, thereby reducing possible polar effects of the *cat* marker. (C) Exact location and genomic context of two Mu*d*Y insertions (*iscR*::Mu*d*Y and *iolR*::Mu*d*Y) in LT2 yielding endogeneous nucleoid reporters.

The Mu*d*K transposon and its delivery system have been described previously [15], and allow the straightforward construction of random *lacZ* translational fusions in *Salmonella* Typhimurium. For our application, we used recombineering [16] to exchange the *lacZYA*-*npt* part of MudK with a *yfp*-*frt*-*cat*-*frt* module, while making sure not to introduce stop codons upstream of the *yfp* open reading frame (Figure 1A). The resulting transposon was designated Mu*d*Y, and can be used for random transposition in *S.* Typhimurium with the potential to create C-terminal translational fusions with the YFP fluorescent reporter protein. Furthermore, the *frt*-flanked chloramphenicol selectable *cat* marker can be readily flipped out by transiently expressing the site-specific Flp recombinase [23], in order to avoid polar effects or to reuse the *cat* marker in further strain engineering (Figure 1B).

In order to validate random transposition and YFP fluorescence, a 6 kb plasmid containing a P_BAD_ promoter was targeted with Mu*d*Y. In total, 10 Mu*d*Y insertions were mapped and all of them yielded different insertion sites in the plasmid, indicative for random transposition of the Mu*d*Y transposon. Furthermore, one of the Mu*d*Y insertions exhibited YFP fluorescence after addition of arabinose (data not shown). The Mu*d*Y transposon is thus able to randomly transpose and can result in YFP fluorescence when the transposon hops in a gene in the correct orientation and reading frame.

### Screening a random Mu*d*Y insertion library for nucleoid reporters

**Figure 2:**
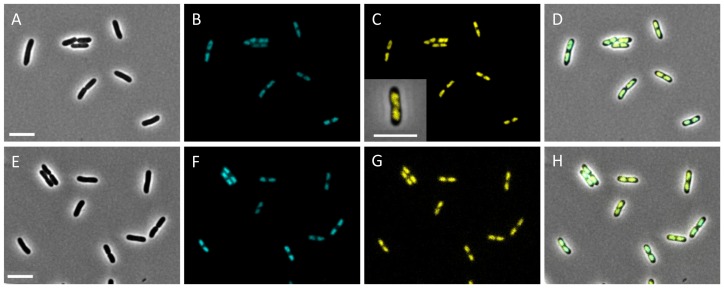
Representative images showing the colocalization of the IscR144::YFP and IolR159::YFP proteins with the DAPI stained nucleoid in LT2 *iscR*::Mu*d*Y (A–D) and LT2 and *iolR*::Mu*d*Y (E–H), respectively. Phase-contrast (A,E), DAPI (B,F), YFP (C,G), and merged (D,H) images are shown. Inset in panel C includes a larger image of an LT2 *iscR*::Mu*d*Y cell showing the apparent helical shape of the nucleoid highlighted by IscR144::YFP. Scale bars correspond to 5 μm.

After having validated the Mu*d*Y transposon and delivery system, a random Mu*d*Y transposition library was constructed in the *S.* Typhimurium LT2 chromosome in order to search for a useful nucleoid reporter. For this, the library of ca. 25.000 clones was first enriched in those clones displaying a clear YFP fluorescence through fluorescence activated cell sorting (FACS; selecting the 0.1% most fluorescent cells). Subsequently, after screening ca. 200 individual clones of this sub-library with fluorescence microscopy, two clones were retained in which YFP fluorescence clearly coincided with the nucleoid as stained with DAPI (Figure 2). Interestingly, the helix-like shape of the nucleoid, most recently described by Hadizadeh Yazdi et al. [24] and Fisher et al. [25], could be observed in a small fraction of cells without using any deconvolution analysis (inset Figure 2). The Mu*d*Y insertion in the two clones could be mapped to the *iscR* (LT2 *iscR*::Mu*d*Y, yielding the IscR144::YFP protein) and *iolR* (LT2 *iolR*::Mu*d*Y, yielding the IolR159::YFP protein) gene, respectively (Figure 1C).

### Validating LT2 *iscR*::*yfp* and LT2 *iolR*::*yfp* nucleoid reporter strains

For further validation, both *iscR*::*yfp* and *iolR*::*yfp* translational fusions were reconstructed *de novo* by fusing the *yfp* moiety (this time with a SGGGG linker) to the penultimate codon of the *iscR* or *iolR* open reading frame on the chromosome, yielding LT2 *iscR*::*yfp* and LT2 *iolR*::*yfp*, respectively. Interestingly, the localization of this reconstructed IolR::YFP fusion protein differed from the original Mu*d*Y mediated one, yielding small nucleoid associated foci instead of the typical “cloudy” appearance of the DAPI stained DNA (Figure S1). In contrast to IolR::YFP, the localization of the reconstructed IscR::YFP fusion did not differ from that of its IscR144::YFP counterpart. Moreover, since the IscR::YFP fusion protein was even more abundantly expressed, LT2 *iscR*::*yfp* was selected for further validation as a nucleoid reporter.

**Figure 3:**
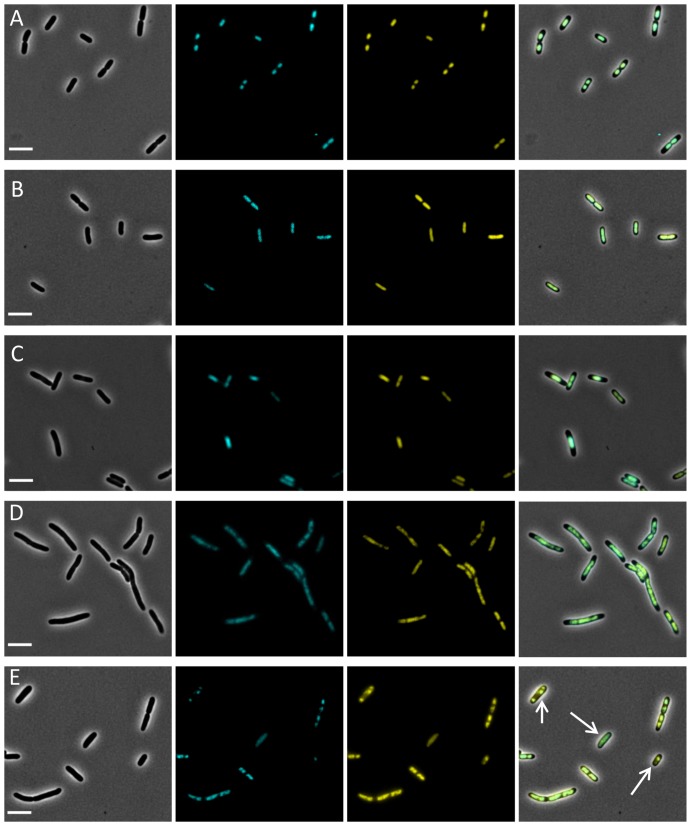
Representative images showing the colocalization of the IscR::YFP protein with the DAPI stained nucleoid in LT2 *iscR*::*yfp* cells stressed with (A) chloramphenicol, (B) rifampicin, (C) nalidixic acid, or (D) mitomycin C. (E) Representative images showing the colocalization of the IscR::YFP protein with the DAPI stained nucleoid in LT2 *iscR*::*yfp recA1 srl-202*::Tn*10* cells, in which compromised RecA function leads to cells with an aberrant nucleoid positioning and morphology (indicated with arrows). For each condition, consecutive panels show phase-contrast, DAPI, YFP, and merged images. Scale bars correspond to 5 μm.

Fluorescence microscopy experiments were conducted using several chemical components that are known to influence nucleoid structuring (Figure 3). In accordance with literature, it was observed that chloramphenicol condenses the nucleoid (Figure 3A) [26] while rifampicin decondenses the nucleoid (Figure 3B) [7]. Nalidixic acid inhibits DNA replication but not cell growth, leading to elongated cells with nucleoid-free regions toward the cell poles (Figure 3C) [27]. Mitomycin C is DNA cross-linking agent, inhibiting DNA replication and ultimately leading to double stranded breaks (Figure 3D) [28], [29]. Furthermore, an LT2 *iscR*::*yfp recA1 srl-202*::Tn*10* derivative was constructed, in which the defective RecA protein often leads to anucleate cells or cells with aberrant nucleoids (Figure 3E) [30]. In all cases, a perfect colocalization was observed when comparing the DAPI channel with the YFP channel.

### Using the IscR::YFP nucleoid reporter to study nucleoid dynamics induced by high hydrostatic pressure stressed growth

**Figure 4:**
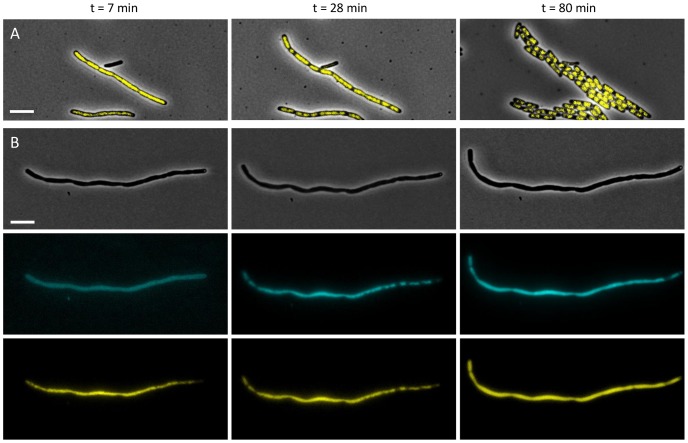
Growth and nucleoid dynamics of HP stressed LT2. (A) Representative images showing cell growth and nucleoid dynamics of HP stressed LT2 *iscR*::*yfp* cells (grown overnight at 40–45 MPa and 37°C) at the indicated time points after pressure release. Merged phase contrast and YFP images are shown. (B) Representative images showing cell growth and nucleoid dynamics of a HP stressed LT2 *iscR*::*yfp* cell (grown overnight at 40–45 MPa and 37°C) at the indicated time points after pressure release in the presence of DAPI and intermittent UV excitation. Phase contrast (top panels), DAPI (middle panels) and YFP (lower panels) images are shown. Scale bars correspond to 5 μm.

In a next step, we were interested to see how nucleoid dynamics were influenced by growth under HP (40–45 MPa, 37°C). The latter condition has been documented to cause excessive filamentation in mesophilic bacteria [10], although this phenotype remains poorly described. Using time-lapse fluorescence microscopy, it was observed that upon pressure release (after growth for 16–20 h at 40–45 MPa, 37°C) most of the filamentous cells initially elongated after which subsequent cell divisions took place leading to a viable microcolony (Figure 4A).

**Figure 5:**
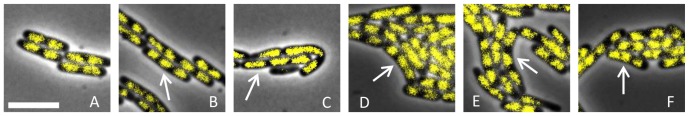
Merged phase contrast and YFP images of growing LT2 *iscR*::*yfp* cells (previously grown overnight at 40–45 MPa and 37°C) after pressure release, showing (A) typical cells with a bilobed nucleoid, and (B–F) occasionally occurring cells with a “trilobed” nucleoid (indicated by arrows). Scale bar corresponds to 5 μm.

Through monitoring the IscR::YFP nucleoid reporter, it could be demonstrated that immediately after HP release the nucleoids appeared as an unsegregated mass that nevertheless quickly started to segregate during cell elongation (Figure 4A). Subsequently, nucleoids were positioned and multiple cell divisions occurred, severing up the filamentous cell and resulting in mostly normal sized cells with the typical bilobed nucleoid appearance. Interestingly, it was observed that during this severing phase, cells with a “trilobed” nucleoid appearance occasionally occurred (Figure 5).

Please note that in contrast to the IscR::YFP reporter, DAPI staining and corresponding UV excitation caused HP treated cells to quickly stop growing, leading to completely inactive cells after 60 min (Figure 4B). The latter observation clearly underscores the importance of non-invasive nucleoid reporters for studying proper nucleoid dynamics in DNA-stressed cells.

### Impact of the IscR::YFP fusion on the fitness of LT2

While examining whether the presence of the IscR::YFP fusion affected the fitness of LT2 *iscR*::*yfp*, a small growth was defected for this strain (and LT2 *iscR*::Mu*d*Y as well) in comparison with LT2 wild-type (Figure S2; in this setup the growth rate of the nucleoid reporter strains is ca. 33% lower than that of the wild type strain). Importantly, since IscR::YFP expression in LT2 *iscR*::*yfp* did not increase basal levels of the DNA damage response (as examined with a previously characterized SOS-reporter strain of LT2 [31]; data not shown), genotoxicity seemed not to be the cause of this growth defect.

Upon further scrutinizing, however, we empirically observed that this growth defect could be mitigated upon construction of an LT2 *iscR*::*yfp-frt-cat-frt* mutant in which the *cat* gene and its promoter were oriented in the same direction as the *iscR* gene (Figure S3), without affecting this strain's capacities as a nucleoid reporter. This latter observation indicates that IscR::YFP expression itself is not toxic *per se*, and suggests that the downstream oriented *cat* promoter is able to counteract the potential polar effects of the *iscR*::*yfp* construct on the remainder of the *isc* operon.

## Discussion

In the search for a non-invasive fluorescent nucleoid reporter, we followed a straightforward rationale in which we first modified the well-characterized Mu*d*K transposon [15] of *S.* Typhimurium LT2 to encode the fluorescent YFP protein such that C-terminal YFP fusion proteins could be obtained after transposon mutagenesis (Figure 1). Random mutagenesis with this transposon (designated Mu*d*Y) and a subsequent screen based on FACS and fluorescence microscopy yielded IolR159::YFP and IscR144::YFP fusion proteins to be specifically located on the nucleoid (Figure 2). While both proteins have previously been reported to be linked with the nucleoid through biochemical and crystallographic studies [32]–[34], this is, to the best of our knowledge, the first time that this has been microscopically validated.

The IolR repressor has been identified as the main regulator of the *myo*-inositol utilization island and binds at least four promoters located on this genomic island of 22.6 kb [32]. The IolR protein belongs to the RpiR family and has two predicted domains: an N-terminal helix-turn-helix-6 (HTH-6) motif and a C-terminal sugar isomerase domain predicted to bind phosphosugars. Comparison of the subcellular localization of IolR159::YFP and IolR::YFP, revealed an interesting difference: While IolR159::YFP colocalized with the typical cloudy appearance of the DAPI stained nucleoid (Figure 2 E–H), IolR::YFP showed distinctive chromosome associated foci (Figure S1). This suggests that lack of the C-terminus in the IolR159::YFP fusion protein, does not abolish the DNA binding ability completely but presumably causes a loss in specificity towards the cognate IolR promoter sites. It is tempting to assume that binding of a specific catabolite of *myo*-inositol to the phosphosugar binding domain of IolR, induces a conformational change in the protein leading to specific recognition of the promoter sites via the HTH-6 motif. However, further investigations are needed to confirm this hypothesis.

The IscR protein has been studied extensively in *Escherichia coli* and was initially discovered as the negative autoregulator of the *isc* (iron-sulfur cluster) operon involved in Fe-S biogenesis [34]. However, it is now known that IscR is a global transcriptional regulator controlling at least 40 genes in 20 predicted operons dispersed throughout the genome [35]. The IscR protein of *S.* Typhimurium has a very high sequence identity (97%) with its *E. coli* homolog and is thus expected to have similar properties. Recently, crystallography data showed that IscR binds as a homodimer to DNA and comprises of two major domains: a DNA binding domain (winged HTH motif) and a dimerization helix [33]. Furthermore, the C-terminal domain of IscR seems not to be involved in the DNA binding properties of IscR, suggesting that a C-teminal YFP-tag will not influence its DNA binding capacity and thus supporting the fluorescence microscopy data obtained with IscR::YFP.

While the IscR-YFP protein could be further validated as a nucleoid reporter through a number of colocalization experiments with the well-established DNA binding dye DAPI (Figure 3), it clearly outperformed this dye in time-lapse experiments that follow up nucleoid dynamics in conditions that are already stressful for the chromosome. In fact, UV/DAPI measurements further aggravated nucleoid stress imposed by HP growth, to the extent that it prevented the proper dynamics to be monitored (Figure 4). In contrast, readout of the IscR-YFP reporter did not impose this bias and allowed us to observe that after HP release the initially unsegregated nucleoid quickly segregated, ultimately leading to a viable microcolony with normal sized cells possessing the typical bilobed nucleoid appearance (Figure 5A). Interestingly, however, during the nucleoid segregation and cell septation process, cells with an unusual “trilobed” nucleoid occasionally emerged (Figure 5B–F), suggesting that during the defilamentation process, coordination of segregation and septation is somehow disturbed.

Please note that our screen for nucleoid reporters was not exhaustive but could in principle be employed to map functional DNA binding domains (or other distinct cellular localization domains) throughout the *Salmonella* proteome, although insertions in some NAPs that are essential in nucleoid organization and regulation might compromise the viability of the corresponding clones and escape detection. Please note that during the preparation of this manuscript other nucleoid reporters such as GFP-Fis And HupA-mCherry have been validated as well, and used to study chromosome organization and dynamics at high resolution [24], [25].

In summary, we have adopted and validated a Mu*d*-based random transposon delivery system to generate translational fusions to the *yfp* reporter gene throughout the *S.* Typhimurium chromosome, and have screened a library of such clones for a suitable nucleoid reporter. The resulting IscR-YFP nucleoid reporter was subsequently used to initiate studies on bacterial nucleoid dynamics resulting from HP stress. More generally, non-invasive fluorescent nucleoid reporters will allow proper nucleoid dynamics to be examined under DNA stressing physiology.

## Supporting Information

Figure S1 **Representative images showing the localization of the IolR::YFP protein on the DAPI stained nucleoid in LT2** ***iolR*** **::** ***yfp*** **cells.** Phase-contrast (A), DAPI (B), YFP (C), and merged (D) images are shown. Scale bar corresponds to 5 μm.
(TIF)

Figure S2 **Growth curves of LT2 wild-type, LT2** ***iscR*** **::Mu** ***d*** **Y and LT2** ***iscR*** **::** ***yfp*** **., with growth monitored as an increase in optical density (OD_630 nm_) in time.** Mean values of 3 independent experiments are shown, with standard deviations being<11%.
(TIF)

Figure S3 **Growth curves of LT2 wild-type and LT2** ***iscR*** **::** ***yfp-frt-cat-frt*** **, with growth monitored as an increase in optical density (OD_630 nm_) in time.** Mean values of three independent experiments are shown, with standard deviations being<8%.
(TIF)
