# Supplementary figures and images for: Isolation and Validation of an Endogenous Fluorescent Nucleoid Reporter in Salmonella Typhimurium
Source: PLoS One. 2014 Apr 2;9(4):e93785. doi: 10.1371/journal.pone.0093785 (PMC3973593; doi:10.1371/journal.pone.0093785)

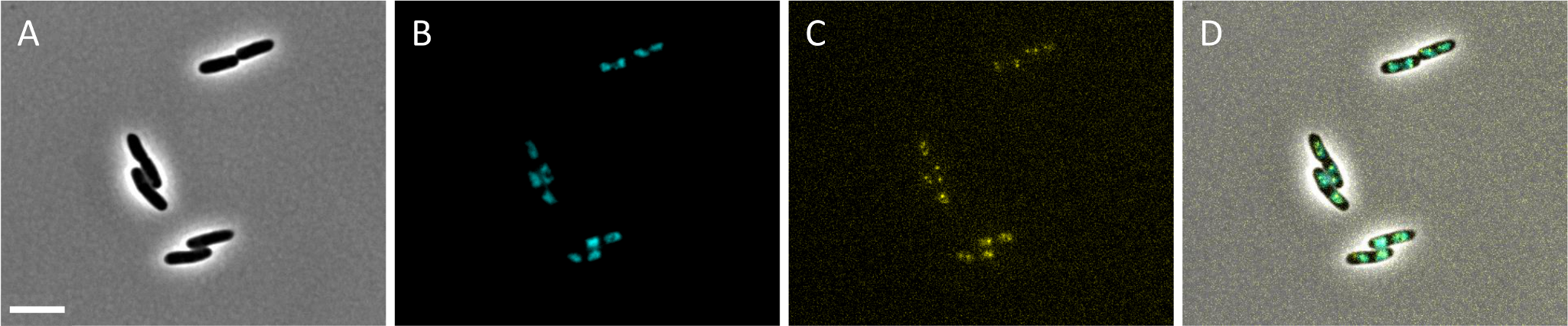

Supplement: Figure S1 — Representative images showing the localization of the IolR::YFP protein on the DAPI stained nucleoid in LT2 iolR :: yfp cells. Phase-contrast (A), DAPI (B), YFP (C), and merged (D) images are shown. Scale bar corresponds to 5 μm. (TIF) [file pone.0093785.s001.tif]

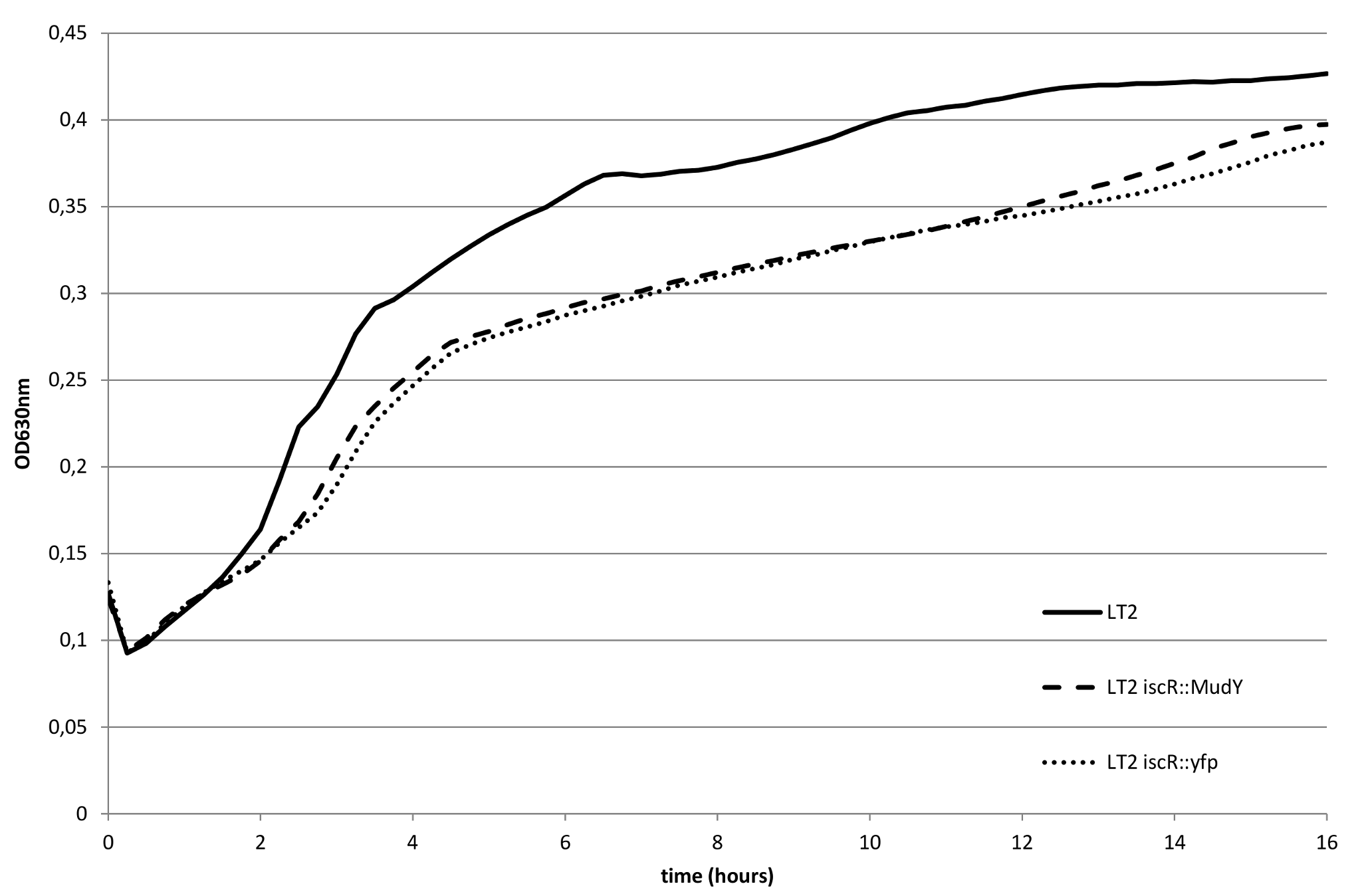

Supplement: Figure S2 — Growth curves of LT2 wild-type, LT2 iscR ::Mu d Y and LT2 iscR :: yfp ., with growth monitored as an increase in optical density (OD630 nm) in time. Mean values of 3 independent experiments are shown, with standard deviations being<11%. (TIF) [file pone.0093785.s002.tif]

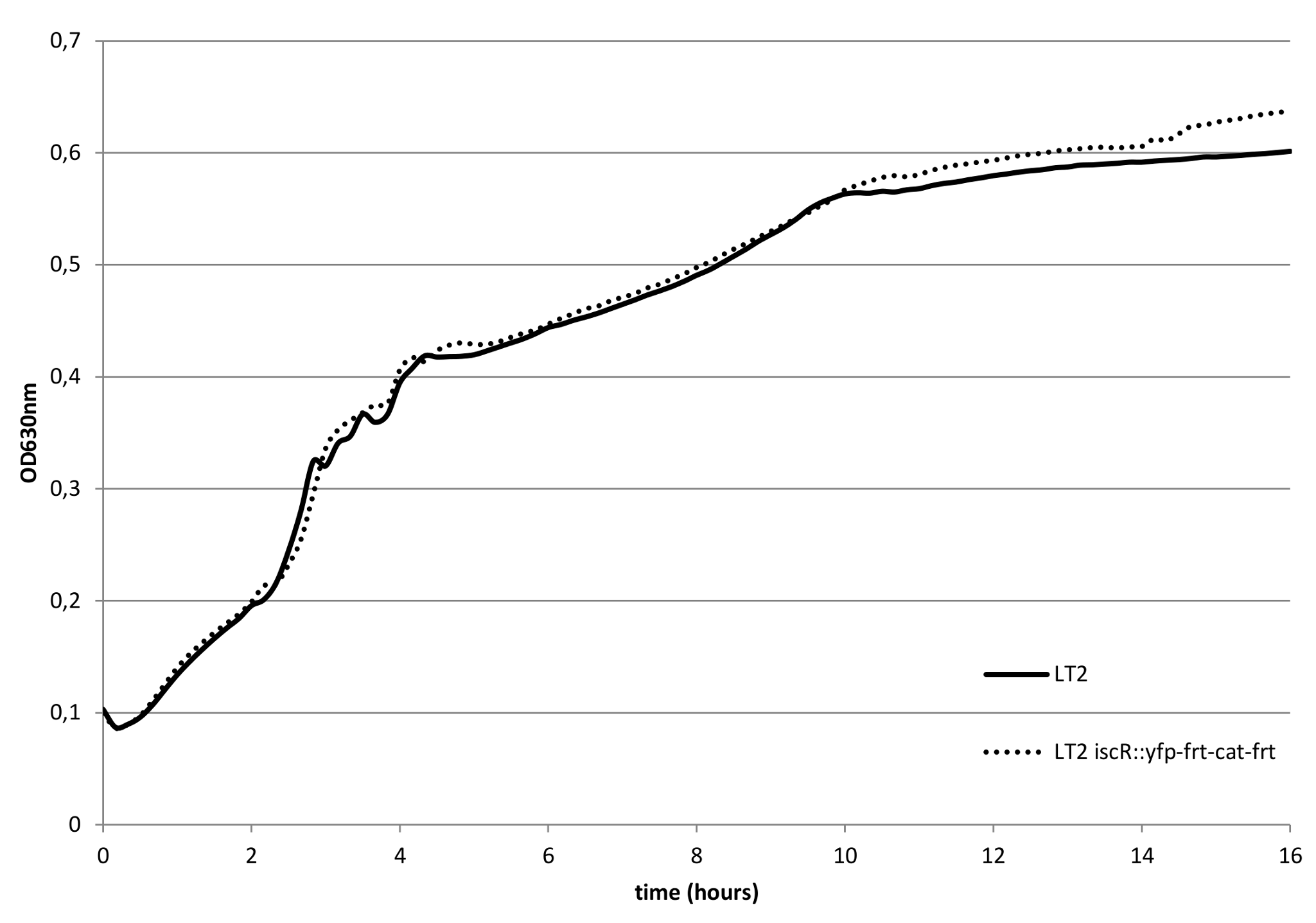

Supplement: Figure S3 — Growth curves of LT2 wild-type and LT2 iscR :: yfp-frt-cat-frt , with growth monitored as an increase in optical density (OD630 nm) in time. Mean values of three independent experiments are shown, with standard deviations being<8%. (TIF) [file pone.0093785.s003.tif]
